# India’s biofuel blending policy presents serious trade-offs with land use, nitrogen emissions and food security

**DOI:** 10.1371/journal.pone.0351419

**Published:** 2026-07-08

**Authors:** Chandan Kumar Jha, Miodrag Stevanović, Prantika Das, Jan Philipp Dietrich, Aline Mosnier, Alexander Popp, Ranjan Kumar Ghosh, Hermann Lotze-Campen

**Affiliations:** 1 Indian Institute of Management Ahmedabad (IIMA), Ahmedabad, India; 2 Potsdam Institute for Climate Impact Research, Member of the Leibniz Association, Potsdam, Germany; 3 FABLE Consortium, Sustainable Development Solutions Network (SDSN), Paris, France; 4 Faculty of Organic Agricultural Sciences, University of Kassel, Witzenhausen, Germany; 5 Department of Agricultural Economics, Humboldt-Universität zu Berlin, Berlin, Germany; Karl-Franzens-Universitat Graz, AUSTRIA

## Abstract

This study examines the environmental and resource implications of India’s 20% ethanol blending mandate, with a focus on its effects on land use, water and fertilizer use, and greenhouse gas emissions. Using a global partial equilibrium model of the land-use sector, MAgPIE, we evaluate scenarios involving different feedstock combinations involving molasses and sugarcane juice. Results reveal that ethanol production from molasses exerts considerable pressure on natural resources due to the high land, water, and fertilizer demands of sugarcane. Conversely, ethanol derived from sugarcane juice proves to be a more sustainable option, requiring less water and fertilizer while generating lower greenhouse gas emissions. Nevertheless, all scenarios present challenges related to food security, through increases in food prices, and resource competition. Sensitivity analysis shows that limited technological progress or constrained trade amplify domestic land, water, and emission burdens. Our findings provide directly usable evidence for designing India’s biofuel roadmap. They can inform (a) the choice of feedstock-mix for achieving blending targets with lower land, water, and nitrogen footprints (b) complementary policies on fertilizer management, irrigation efficiency, and trade; and (c) the timing and scale-up of diversified and second-generation feedstocks. Overall, the study highlights that careful management of feedstock portfolios and supporting agricultural policies is essential for aligning India’s ethanol expansion with long-term climate, food security, and resource sustainability goals.

## 1 Introduction

The Paris Agreement aims to limit global temperature rise to well below 2°C, with efforts to cap it at 1.5°C [1]. Bioenergy is expected to play a crucial role in emission reductions, offering potential for low- or even net-negative greenhouse gas (GHG) emissions across electricity, heat, and transportation fuels [2–5]. India, as a part of its updated climate pledge to the UNFCCC, has committed to reducing the emissions intensity of GDP by 45% by 2030 from 2005 levels. With net-zero targets set for 2070, decarbonizing the transport sector responsible for 12.9% of energy related GHG emissions and 9.7% of total emissions (excluding LULUCF) in 2016 – is a critical priority. The sector currently consumes 19% of the final energy and 50% of India’s oil, and demand is projected to surge as urbanization, incomes and living standards rise, making India the fastest growing energy market by 2040.

To address these challenges, policymakers are promoting cleaner fuels, particularly biofuels, to reduce emissions intensity and decouple transport sector growth from high emissions. India’s biofuel policy 2018 mandates 20% ethanol and 5% biodiesel blending by 2030, a target recently advanced to 2025. The policy emphasizes cultivating non-edible oilseed plants on wastelands to avoid compromising food security and permits ethanol production from surplus food grains to support government goal of doubling farmers’ income. The updated policy expands the scope to include first generation feedstocks like sugarcane juice, sugar beet, sweet sorghum, and damaged grains along with second-generation feedstocks, such as agricultural residues and municipal waste. A life cycle assessment (LCA) of sugarcane-based biofuel production in India shows that ethanol produced from cane or molasses can achieve substantial greenhouse-gas reductions, often up to 70–76% lower than gasoline primarily because sugar mills use bagasse for process energy, reducing fossil fuel demand [6]. Integrated farm-to-gate LCAs further indicate that although sugarcane ethanol delivers strong climate and energy benefits, it also exhibits a high-water footprint, making water scarcity a key sustainability concern in major producing regions [7].

However, biofuels pose several sustainability challenges due to their indirect effects on land use change, GHG emission, food security, and ecosystem services [8,9]. Among the significant concerns is the impact of crop-based biofuel expansion on food prices, which exacerbates food security risks [10–12]. Moreover, the diversion of agricultural land for biofuel production leads to deforestation and reduces food availability [13–15]. Empirical research also highlights additional land-related challenges – including conflicts, land grabbing for biofuel projects, loss of biodiversity, and adverse impacts on water and soil resources [16–18]. Questions also remain about biofuels effectiveness in reducing GHG emissions. While some studies suggest a high potential for bioenergy, others argue that alternative land uses such as reforestation, may offer more effective climate mitigation [19–21]. Critics highlight that the carbon costs associated with bioenergy production may outweigh its environmental benefits [22,23].

Past studies have evaluated the impacts of India’s biofuel mandates using diverse methods, each highlighting critical considerations for sustainable biofuel development. For instance, Saravanan et al. [24] conducted a systematic literature review emphasizing the need for holistic biofuel policies that balance environmental objectives with food security and socio-economic impacts. Ravindranath et al. [25] used two factor interaction calculations to analyse ethanol and biodiesel targets, recommending the use of wastelands for biofuel crops but cautioning against insufficient consideration of increasing food and feed crop demands. Khanna et al. [26] employed a price-endogenous multi-market equilibrium model to explore how biofuel policies influence market dynamics, revealing trade-offs between biofuel targets and food security due to price and demand shifts. Sakthivel et al. [27] focused on ethanol production sectoral contributions, underscoring the importance of aligning production targets with sustainability and highlighting land and resource constraints as key challenges.

While these studies provide valuable insights, they lack an integrated assessment of land-use changes across agricultural sub-sectors. A comprehensive evaluation must incorporate dynamic economic and demographic trends in agricultural consumption, local biophysical factors affecting production, and potential international trade implications. Such an analysis would need to integrate various scales while accounting for diverse land qualities and technological differences. As India’s population and economy grow, biofuel production becomes increasingly important for energy security, but it also introduces trade-offs between energy and food crops, alongside mounting pressure on natural resources and livestock feed.

In this context, we assess the implications of India’s biofuel blending mandate, focusing on sugarcane-based feedstocks and key food and land use indicators through 2050. Using the Model of Agricultural Production and its Impact on the Environment (MAgPIE) [28], a global spatially explicit agricultural sector model, we analyse the impacts on land use, GHG emissions, food prices, agricultural water use, fertilizer use, and international trade. The analysis compares results across four scenarios involving different feedstock options, based on molasses and sugarcane juice. The study also presents a sensitivity analysis of differential impacts under varying trade and technological conditions.

## 2 Methodology and scenario description

### 2.1 Brief description of MAgPIE

This study employs the MAgPIE model to analyze the effects of India’s biofuel blending mandate [28–31]. MAgPIE minimizes the cost of meeting the demand for agricultural products while considering socio-economic and biophysical constraints in agricultural production systems. The model operates at a global level with 12 socio-economic regions, treating India as a distinct region. It optimizes agricultural production, land use, irrigation water use, and carbon stock variations, using spatially explicit biophysical data on 0.5°x0.5° geographical grid. These data, derived from the grid-based dynamic vegetation model LPJmL [32], includes simulations of irrigated and rainfed crop yields (S6 Fig in S1 File), terrestrial carbon stocks, and water availability. The model determines optimal land use patterns for cropland, pastures, forests, and other natural vegetation, as well as optimal investment rates in yield-increasing technological change [33], investment in land expansion and irrigation infrastructure. The model integrates international trade to ensure global demand for food, feed, seed, processing, and bioenergy is met, while accounting for historical trade patterns and regional economic competitiveness in agricultural trade flows. Prices are represented through a food price index calculated using the Paasche price index methodology, which weights current prices based on food baskets from the same time period [34]. Shadow prices are used to estimate the marginal cost of increasing output, offering insights into cost dynamics as production scales. Additional details about the model and its underlying assumptions can be found in Sect 1 and S1 Fig of the S1 File.

### 2.2 Bioethanol modelling in MAgPIE

MAgPIE simulates both first- and second-generation bioenergy crop production with the former relying on food crops and the latter on dedicated herbaceous and woody lignocellulosic crops [35]. The model assumes that primary agricultural products can be processed into secondary products such as sugar and ethanol. Demand for first-generation bioenergy is defined at the regional level based on existing biofuel policies [36]. In India, current bioethanol production predominantly depends on first-generation feedstocks derived from conventional food crops, particularly sugarcane, with maize, other crops, and crop residues playing a smaller role. Therefore, this analysis focuses on processing sugarcane and its by-products for bioenergy. Analysis of second-generation biofuels is not considered in this study.

To evaluate the Indian biofuel blending case, we set the blending target to be achieved by 2030, in alignment with the India’s National Bioenergy Policy 2018. Ethanol demand was estimated by first projecting India’s total petroleum demand for 2030 and 2050 using time series data from 1990 to 2018 [37]. Based on these projections, the bioethanol required to meet the 20% blending target by 2030 was calculated (S2 Fig in S1 File). Bioethanol demand is projected to rise from 0.4 GJ/year in 2015–788 GJ/year by 2030, and further to 1838 GJ/year by 2050 (see S2 Fig in S1 File).

### 2.3 Parameterisation of bioethanol processing

This analysis examines two primary feedstock pathways for bioethanol production, molasses and sugarcane juice, to estimate the ethanol requirements and corresponding sugarcane demand necessary to achieve India’s biofuel blending targets by 2050 (S5 Fig, S1 File). These estimates rely on conversion rates from primary to secondary products as represented within the MAgPIE modelling framework. The parameterization of sugarcane-based ethanol production is presented in S3 Fig (S1 File). India-specific conversion factors are applied to derive the demand for molasses and sugarcane juice from sugarcane, followed by their respective conversion to ethanol [38,39]. In MAgPIE, molasses is treated as a by-product of sugar refining, with a yield of 0.045 t molasses per tonne of sugarcane, consistent with FAO and National Sugar Institute estimates [40,41]. Molasses-based ethanol production is modelled at 0.187 t ethanol per tonne of molasses, aligning with standard Indian industry yields of roughly 235 L ethanol per tonne of molasses [41] (Karnataka Economic Analysis, 2021). Ethanol production from sugarcane juice is assumed to yield 0.135 t ethanol per tonne of sugarcane, representing an upper-bound technological efficiency relative to current empirical estimates of 70–81 L ethanol per tonne of cane [42,43].

### 2.4 Scenario description

To assess the impacts of the biofuel blending mandate, we developed five scenarios, including a baseline, each defined by varying shares of molasses and sugarcane juice as feedstocks. In all scenarios (excluding the business-as-usual (BAU) scenario), it is assumed that India achieves the 20% blending target by 2030, with bioethanol demand continuing to rise linearly through 2050. We evaluate the changes in key indicators across alternative scenarios from 2020 to 2050.

#### 2.4.1 Business-as-usual (BAU).

The BAU scenario serves as the baseline, characterized by an existing demand for first-generation bioenergy (S4 Fig in S1 File) without any policy mandate targeting its use. In this scenario, bioenergy consumption is primarily limited to traditional uses, such as heating. The underlying assumption of the BAU scenario, including agricultural and food demand future projections, is based on the SSP2 ‘middle-of-the-road’ scenario [31,44]. This scenario represents a continuation of current social, economic, and technological trends, with no additional policy interventions beyond India’s Nationally Determined Contributions (NDCs). In MAgPIE, the NDCs are represented by India’s afforestation target under the Paris Agreement, assuming deforestation halts post-2010 and an additional 32 million hectares of afforestation is achieved by 2030. Population in India under SSP2 is projected to reach 1.59 billion by 2030 and 1.63 billion by 2050, while per capita income rises from $9,235 (US$05 PPP/year) in 2030 to $16,789 (US$05 PPP/year) in 2050. The climate change trajectory follows Representative Concentration Pathway (RCP) 7.0, associated with a potential 4°C increase in mean global temperature by the end of the century. This trajectory leads to climate-related impacts on crop yields, water availability, and terrestrial carbon, all of which are incorporated into the MAgPIE modeling framework.

#### 2.4.2 AllMolasses.

This scenario adopts the same underlying assumptions as the BAU scenario, except for the first-generation bioenergy demand and its feedstock sources. It aligns with India’s NBP 2018, targeting a 20% bioethanol blending mandate by 2030. In this scenario, it is assumed that the mandated ethanol demand is met exclusively through molasses feedstock. Trade and technological change in this scenario are endogenous, enabling the model to achieve the food and fuel demand through yield improvements and commodity imports.

#### 2.4.3 Mix3070.

Like the AllMolasses scenario, this scenario maintains the assumption of meeting the target demand for first-generation bioenergy. However, it introduces a differentiated feedstock share for ethanol production, i.e., 30% of the ethanol is derived from molasses and the remaining 70% is produced directly from sugarcane juice directly through distillation. All other assumptions remain the same as in AllMolasses scenario.

#### 2.4.4 Mix1090.

In this scenario, it is assumed that 10% of the ethanol is sourced from molasses, with the remaining 90% derived directly from sugarcane juice. All other assumptions are consistent with those in the AllMolasses scenario.

#### 2.4.5 AllSugarjuice.

This scenario assumes that all ethanol required to meet the mandate is derived directly from the sugarcane juice. All other assumptions are identical to those in the AllMolasses scenario.

## 3 Results

### 3.1 Impacts on land use

**Fig 1:**
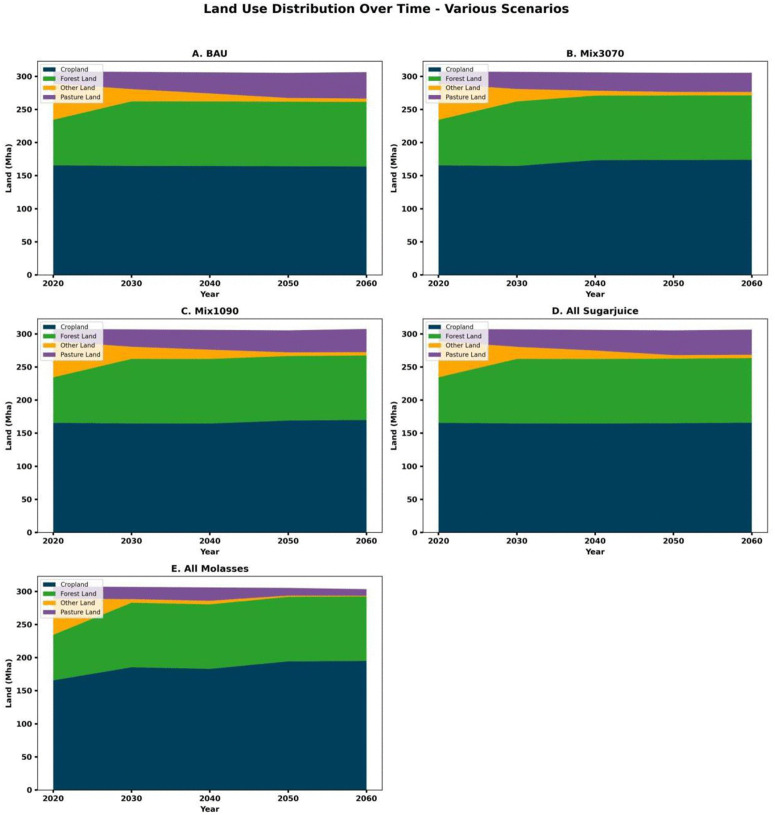
Projected land cover (in Mha) for cropland, other land, pasture land, and forest land till 2050 for different scenarios. The figure illustrates land cover projections under various scenarios, showing an increase in cropland area in the AllMolasses and Mix3070 scenarios. This expansion is attributed to the growing demand for sugarcane cultivation by 2050. In these scenarios, the additional cropland area primarily comes from the conversion of other land and pasture land.

Changes in land use across alternate scenarios are presented in Fig 1. In the BAU scenario, where no ethanol targets are set, the total cropland area is projected to decline slightly by 1% by 2050. Forestland, however, expands significantly, by 42% across all scenarios, driven by India’s NDC commitments, which require increased forest cover by 2030 and restrict forest-to-cropland conversions. Pastureland expands significantly, growing by 41% from 18.8 million hectares (Mha) to 26.5 Mha by 2030, followed by a remarkable 102% surge to 38 Mha by 2050. Meanwhile, “other land” categories, including barren and unused lands, decrease by 90% to make way for the expansion of forests and pastures.

Under the ‘AllMolasses’ scenario, meeting the ethanol blending mandate drives a significant expansion in sugarcane cultivation, increasing from 4 million hectares (Mha) in 2020–83 Mha by 2030, and further to 101 Mha by 2050. Consequently, cropland area grows by 9% to 11%, rising from 165 Mha in 2020–180 Mha by 2030 and 184 Mha by mid-century. In comparison, the ‘Mix3070’ and ‘Mix1090’ scenarios, which diversify feedstocks to include sugarcane juice, there is moderate cropland expansion to just 4% and 1% above 2020 levels, respectively. These mixed scenarios reduce the required sugarcane area by 47% (’Mix3070’) and 80% (’Mix1090’) relative to the ‘AllMolasses’ pathway, demonstrating the benefits of diversification in mitigating sugarcane-driven land pressure.

The increase in cropland across all scenarios is primarily achieved by converting “other land,” which diminishes by 90% compared to 2020. However, the heavy reliance on sugarcane in the ‘AllMolasses’ scenario exerts substantial pressure on pastureland, reducing it by 52% compared to BAU by 2050. In contrast, the ‘Mix3070’ and ‘Mix1090’ pathways maintain much higher pastureland levels, 69% and 94% greater, respectively, than in the ‘AllMolasses’ scenario. This is critical as rising incomes drive higher livestock product demand, necessitating more pastureland for animal feed.

The findings reveal that meeting the biofuel mandate exclusively with molasses feedstocks requires a significant expansion of sugarcane cultivation, approximately 21% to 25% higher than the BAU scenario. While the mixed feedstock scenarios reduce sugarcane acreage, the area required by 2050 remains 5–12 times greater in Mix1090 and Mix3070, respectively, compared to the BAU in AllSugarjuice scenario. This raises concerns about the potential emergence of large-scale sugarcane monocultures, with associated environmental and economic implications.

Advances in crop productivity, supported by technological innovation and improved irrigation infrastructure, play a crucial role in alleviating land-use pressure. By 2050, the technological advancements required to boost sugarcane yields in the ‘AllMolasses’ scenario are 133% higher than in BAU. In contrast, productivity growth requirements in ‘Mix3070’ and ‘Mix1090’ are more manageable, at 29% and 9% above BAU levels (S4 Fig in S1 File). Furthermore, the technological requirements in the mixed scenarios are 45% (’Mix3070’) and 53% (’Mix1090’) lower than in the ‘AllMolasses’ pathway.

In summary, ethanol pathways that prioritize a higher proportion of sugarcane juice as feedstock significantly reduce land-use changes. Even without substantial improvements in productivity, these diversified approaches offer a more sustainable and balanced path for meeting India’s ethanol mandates while minimizing environmental trade-offs.

### 3.2 Impacts on production and trade

**Fig 2:**
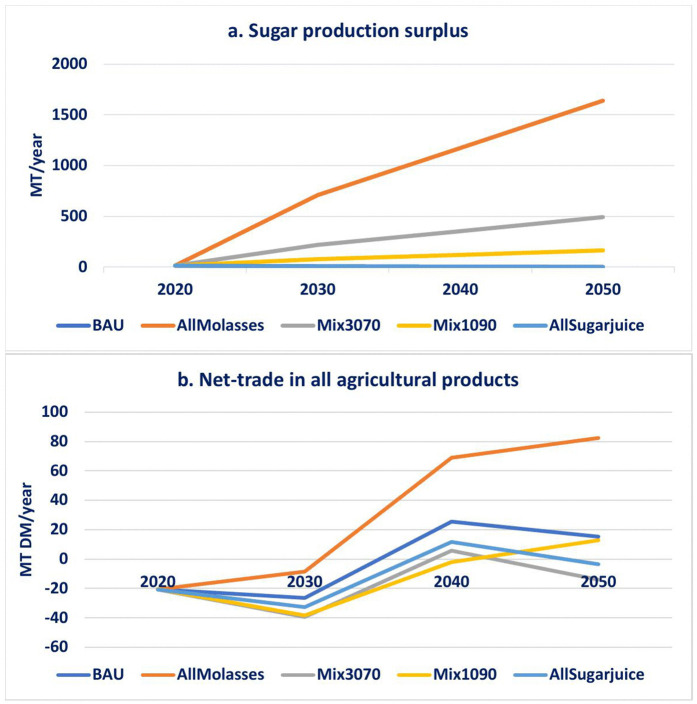
The figure a. shows the overproduction of sugar (in Mt yr^-1^) across the scenario between 2020 to 2050. The overproduction of sugar evolves with the share of sugarcane-based molasses route and sugar juice. With decreasing share of sugarcane-based molasses, the overproduction of sugar decreases. The figure b. shows net trade (Export – Import) for all agricultural products, indicated in Mt yr^-1^ across the scenarios between 2020 and 2050.

Fig 2a and 2b illustrate the implications of various scenarios on sugar production and trade dynamics. In the BAU scenario, the model effectively balances supply and demand, maintaining market equilibrium. However, biofuel mandate scenarios that rely on molasses as an ethanol feedstock lead to a substantial increase in sugar output, because sugar production is linked to sugarcane processing through fixed processing coefficients, which define sugar as a by-product of molasses-based ethanol production. Within MAgPIE partial equilibrium framework, market clearing is achieved by satisfying exogenously specified sugar demand; however, additional sugar generated as a by-product of molasses-based ethanol production does not necessarily find a domestic market. The model allows this excess production either to be exported—subject to historical trade shares and comparative advantage—or to remain as regional production surplus when export capacities are constrained. Given India’s historically limited sugar exports and trade restrictions, only a fraction of the surplus can be absorbed through international markets, resulting in large residual surpluses in high-molasses scenarios. Under the ‘AllMolasses’ pathway, sugar surpluses are projected to reach 710 million tonnes (Mt) by 2030, more than doubling to 1,640 Mt by 2050, highlighting the unsustainable nature of this approach in managing excess production.

Scenarios that shift ethanol production toward direct utilization of sugarcane juice, such as ‘Mix3070’ and ‘Mix1090,’ demonstrate a marked improvement in mitigating overproduction. The ‘Mix3070’ scenario reduces annual sugar surpluses to 218 Mt in 2030 and 492 Mt in 2050, translating to reductions of approximately 69% and 70%, respectively, when compared to the ‘AllMolasses’ pathway. The ‘Mix1090’ scenario performs even better, curbing surpluses to 77 Mt by 2030 and 164 Mt by 2050, representing reductions of 89% and 90%, signalling a more effective strategy for addressing systemic overproduction issues.

The most transformative impact arises from the governments policy allowing ethanol production directly from sugarcane juice. This intervention significantly reshapes production dynamics, as evident in the ‘AllSugarjuice’ scenario, where sugar surpluses dramatically decline to 7 Mt by 2030 and are eliminated by 2050. This scenario underscores the critical role of policy shifts in aligning production outputs with market demand and alleviating pressure on sugar prices, presenting a sustainable pathway for managing sugar sector challenges.

Under the AllMolasses scenario, net trade remains stable at baseline levels in 2030 but rises to 35 Mt yr ⁻ ¹ by 2050, marking a significant 100% growth in exports of all agricultural products over the period. This increase is largely driven by technological advancements that enhance crop yields, allowing for surplus production despite land pressures from sugarcane cultivation.

However, scenarios prioritizing ethanol production from sugarcane juice, such as Mix3070 and Mix1090, reveal a contrasting dynamic, with a significant shift toward rising imports for all agricultural products. In the Mix3070 scenario, net trade declines to −71 Mt yr ⁻ ¹ in 2030, improving to −52 Mt yr ⁻ ¹ by 2050, showing a 27% reduction in net imports over the two decades. The Mix1090 scenario shows a similar trajectory, with net trade dropping to −68 Mt yr ⁻ ¹ by 2030, then recovering to −18 Mt yr ⁻ ¹ by 2050, a substantial 74% decrease in net imports. These shifts suggest that diverting sugarcane juice for ethanol production exacerbates land use pressures, reducing the availability of land for other crops and thereby increasing reliance on imports to meet domestic food demand.

The ‘AllSugarjuice’ scenario highlights the most pronounced impact of land use changes. Net trade will decline to −61 Mt yr ⁻ ¹ by 2030, improving modestly to −34 Mt yr ⁻ ¹ by 2050, reflecting a 44% reduction in net imports over this period. While this pathway effectively addresses domestic sugar overproduction, the additional land required for sugarcane cultivation significantly curtails the production of other agricultural commodities, increasing import dependency to fill the resulting supply gaps.

### 3.3 Impacts on food prices

**Fig 3:**
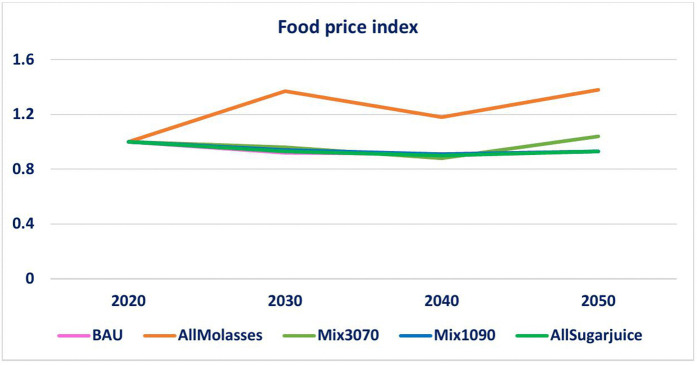
Changes in food price index (index 2020 = 1) between 2020 and 2050 under the (a) Baseline, (b) AllMolasses (c) Mix3070 (d) Mix1090 and (e) AllSugarjuice scenario. Food price index in the model follows the Paasche price index by weighing current prices based on food baskets in the same period.

The oversupply of sugar plays a significant role in driving up food prices, primarily through land-use competition. In the AllMolasses pathway, the food price index increases sharply, rising by 49% above BAU levels by 2030 and 48% by 2050 (Fig 3). This inflationary pressure is largely due to the additional land required for sugarcane cultivation, which competes with land needed for essential food crops such as cereals, oilseeds, and pulses. Furthermore, the costs of land conversion and the technological advancements required to maintain high productivity further exacerbate the price increase.

Scenarios that allocate a larger share of sugarcane juice for ethanol production, such as Mix3070 and Mix1090, reduce the need for expanded sugarcane acreage, alleviating land-use pressures. In the Mix1090 scenario, for example, food price increases are minimal, with the food price index remaining closer to BAU levels. This shift demonstrates how adjusting the feedstock mix can stabilize food prices by reducing the competition for land. The impact on food prices becomes more pronounced as the share of molasses in ethanol production increases. In the Mix3070 scenario, where molasses represents a moderate share, food price inflation is noticeable but relatively contained. However, in the All Molasses scenario food prices rise substantially, that is, 60% above BAU levels by 2030 and 42% by 2050. This significant increase is driven by the direct competition for land between sugarcane and food crops, which necessitates additional land expansion and incurs costs related to land conversion and technological upgrades. In contrast, the AllSugarjuice scenario leads to the smallest increase in food prices. The food price index rises by just 1% above BAU levels by 2030 and aligns with the no-biofuel policy scenario by 2050, highlighting the minimal land-use competition when sugarcane juice is prioritized for ethanol production.

### 3.4 Impacts on water and fertilizer use

**Fig 4:**
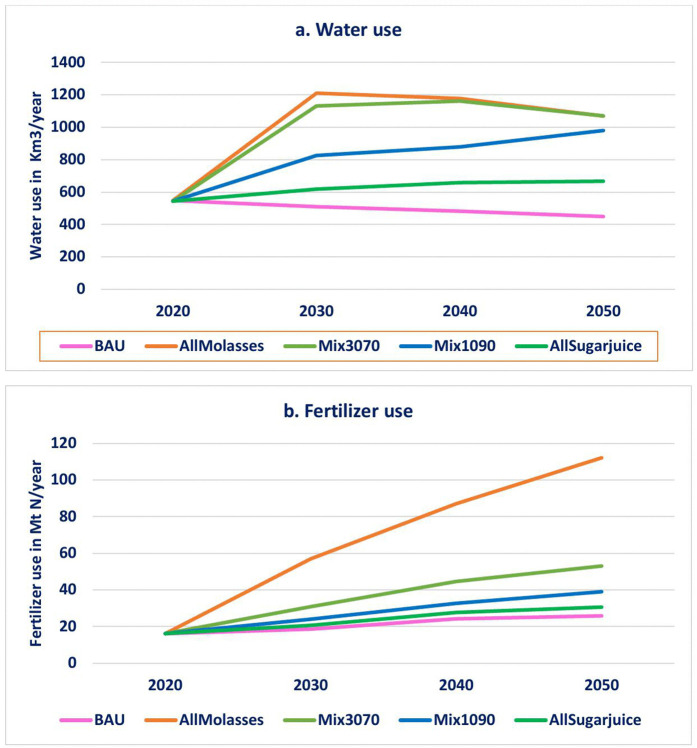
Changes in a. Water Use (Km3 yr^-1^) and b. Fertilizer Use (in Mt N yr^-1^) across alternative scenarios up to 2050. The results indicate that scenarios with higher sugarcane demand, such as AllMolasses and Mix3070, lead to significant increases in water and fertilizer use compared to the baseline. In contrast, the Mix1090 and AllSugarjuice scenarios, which feature a greater share of ethanol production from sugarcane juice and reduced sugarcane demand, exhibit comparatively lower water and fertilizer use.

**Fig 6:**
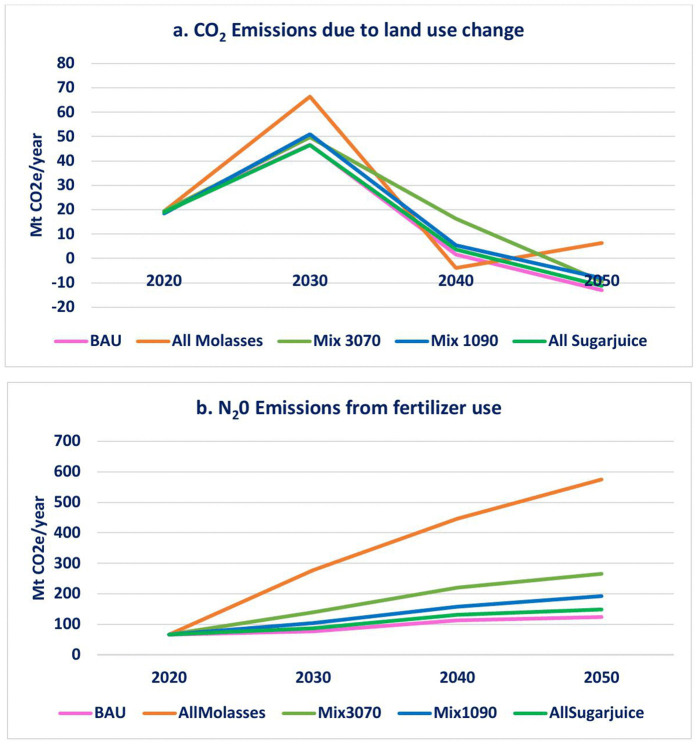
Projected GHG Emissions (Mt CO₂e yr ⁻ ¹) from (a) Land Use Change and (b) Nitrogen Use across Scenarios up to 2050. The figure illustrates those scenarios with a higher share of molasses-based ethanol production result in increased GHG emissions from both land use change and fertilizer application. This trend is driven by the elevated sugarcane cultivation required to meet the higher demand, leading to greater emissions associated with land conversion and nitrogen overload from the increased use of inorganic fertilizer.

The environmental effects on water (Fig 4a) and fertilizer use (Fig 4b) are presented in figure. In the BAU scenario, reduced agricultural productivity under the RCP 7.0 climate scenario is projected to lower water demand, resulting in a 7% decline in agricultural water use by 2030 and an 18% decline by 2050 relative to 2020 levels. However, alternative ethanol production pathways lead to substantial increases in water usage. Compared to BAU, water consumption rises by 21–118% in 2030 and 49–138% in 2050 across the four scenarios, with the ‘AllSugarjuice’ scenario requiring the least water and the ‘AllMolasses’ scenario the most (Fig 6a).

Shifting the ethanol feedstock mix toward sugarcane juice appears to ease water usage compared to the ‘AllMolasses’ scenario, as water use decreases by 7–49% in 2030 and 12–45% in 2050 across the other scenarios. This suggests that increasing the share of sugarcane juice in ethanol production could help mitigate water demand to some extent. However, relative to 2020 levels, water use in all sugarcane-based ethanol scenarios remains significantly higher, ranging from 14–121% by 2030 and 23–95% by 2050. Despite the comparatively lower water demand in the ‘AllSugarjuice’ scenario, the high consumption levels across all pathways raise serious concerns about the sustainability of water resources.

The primary driver of these concerns is the water-intensive nature of sugarcane cultivation in India, which requires approximately 20 million Liters per hectare, with around 80% of this demand met through groundwater extraction. This heavy reliance on groundwater exacerbates sustainability challenges, particularly in regions already facing water scarcity. Studies show that sugarcane in tropical regions like India has a disproportionately large blue water footprint (surface and groundwater use) compared to its green water footprint (rainwater use) [45]. Although sugarcane juice-based ethanol production reduces land requirements relative to molasses-based approaches, it offers limited relief for water stress. The substantial water demands of sugarcane cultivation could further strain resources, potentially reducing water availability for other crops and essential agricultural activities. These findings underscore the need for a more sustainable approach to ethanol production, balancing energy goals with water resource management.

Fertilizer usage (Fig 4b) also poses a significant challenge. Under the BAU scenario, fertilizer application is projected to increase by 60% by 2050 compared to 2020 levels, driven by rising food demand. In the ‘AllMolasses’ scenario, fertilizer use experiences an extraordinary rise of 600%, surging from 16 Mt N in 2020–112 Mt N by 2050 due to the large-scale expansion of sugarcane cultivation. The ‘Mix3070’ and ‘Mix1090’ pathways moderate this growth, with fertilizer use increasing by 231% and 144%, respectively, to reach 53 Mt N and 39 Mt N by 2050. In the ‘AllSugarjuice’ scenario, fertilizer use rises more modestly, increasing by 31% to 21 Mt N by 2030 and by 94% to 31 Mt N by 2050. Despite these differences, fertilizer use in 2050 remains significantly higher than BAU levels, exceeding it by 333% in ‘AllMolasses,’ 105% in ‘Mix3070,’ 51% in ‘Mix1090,’ and 10% in ‘AllSugarjuice’ (Fig 4b).

This surge in fertilizer application also results in a substantial increase in nitrogen surplus, exacerbating environmental degradation. Compared to BAU, nitrogen surplus is projected to rise by 278% in the ‘AllMolasses’ scenario, 90% in ‘Mix3070,’ 43% in ‘Mix1090,’ and 10% in ‘AllSugarjuice,’ highlighting the environmental costs of intensified sugarcane cultivation for ethanol production.

### 3.5 Impacts on GHG emissions

**Fig 5:**
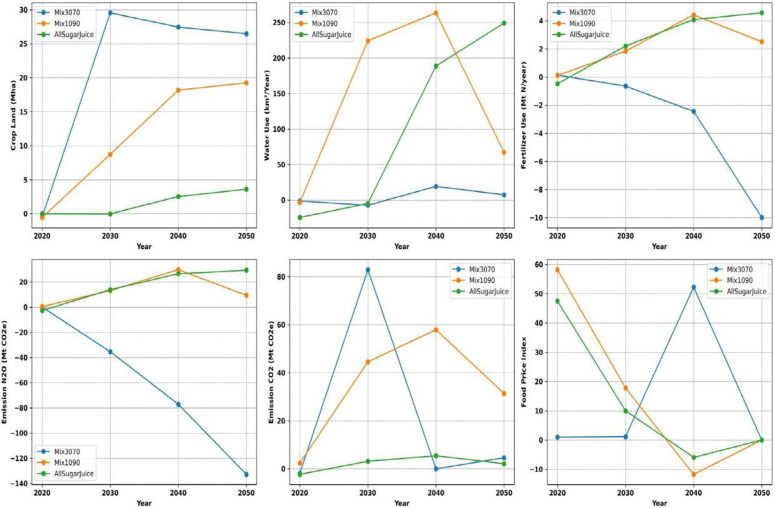
The figure shows the change in Crop Land (in Mha), Water Use (in Km^3^ Yr^-1^), Fertilizer Use (in Mt N yr^-1^), Emission due to Nitrogen Use (In Mt CO_2_e yr^-1^), Emission due to Land Use Change (In Mt CO_2_e yr^-1^) and change in Agriculture Prices under Mix3070, Mix1090 and AllSugarjuice scenarios in comparison to the Mix3070Exo, Mix1090Exo and AllSugarjuiceExo scenario. This represents the additional pressure related to resource and environmental impacts when we have restricted technological change and trade to BAU levels.

In the BAU scenario, emissions from land-use change (Fig 5a) increases to 47 Mt CO₂e annually by 2030 due to cropland expansion. By 2050, these emissions shift to a net removal of −13 Mt CO₂e per year, primarily driven by afforestation efforts that offset earlier emissions. Nitrogen-related emissions, primarily from fertilizer use, exhibit a steady upward trend under BAU, rising from 77 Mt CO₂e annually in 2030–124 Mt CO₂e by 2050. This underscores the significant contribution of fertilizer application to overall emissions.

In the ‘AllMolasses’ scenario, land-use change emissions reach 6 Mt CO₂e annually by 2050, a 148% increase compared to BAU. This increase is attributed to the conversion of land previously allocated to natural vegetation into sugarcane fields for ethanol production. Conversely, scenarios with a higher reliance on sugarcane juice for ethanol production ‘Mix3070’ and ‘Mix1090’ result in net carbon removals of 9 Mt CO₂e and 8 Mt CO₂e annually, respectively, by 2050 (Fig 6a). The ‘AllSugarjuice’ scenario presents a distinct trend. Emissions from land-use change rise to 46 Mt CO₂e annually by 2030, like BAU, but transition to a net removal of −11 Mt CO₂e per year by 2050 due to afforestation. Nitrogen-related emissions (primarily nitrous oxide) from the use of soil organic fertilizers increase to 87 Mt CO₂e annually by 2030 and further to 149 Mt CO₂e by 2050. This represents a more substantial impact on nitrogen emissions compared to BAU, emphasizing the trade-offs of relying exclusively on sugarcane juice for ethanol production.

Nitrogen-related emissions also vary across other scenarios (Fig 6b). In the ‘AllMolasses’ pathway, nitrous oxide emissions escalate to 575 Mt CO₂e annually by 2050, driven by extensive sugarcane acreage and intensive nitrogen application. By contrast, in the ‘Mix3070’ and ‘Mix1090’ scenarios, emissions are significantly reduced to 266 Mt CO₂e and 192 Mt CO₂e per year, respectively. However, these emissions remain higher than BAU by 114% (’Mix3070’) and 55% (’Mix1090’), underscoring the ongoing challenge of managing nitrogen pollution. India’s nitrogen application rates, ranging from 150 to 400 kg N ha ⁻ ¹ annually, are substantially higher than those observed in countries like Brazil [46,47]. This highlights the need for more efficient fertilizer management practices to mitigate the environmental impacts associated with intensive nitrogen use. In summary, while increasing the share of sugarcane juice in ethanol production, as in the ‘AllSugarjuice’ scenario, reduces land-use change emissions, it results in higher nitrogen-related emissions compared to BAU. Effective fertilizer management and optimized land-use strategies are critical for minimizing emissions and ensuring sustainable biofuel production pathways.

### 3.6 Sensitivity analysis

The ability to meet biofuel mandates with minimal environmental and resource stress is critically dependent on advancements in agricultural technology and international trade. The results discussed above assume endogenous technological change and trade. These assumptions allow the model to meet the additional demand requirements of crop commodities for food and fuel through yield improvement, resource efficiency and imports. We now examine the sensitivity of our results to this assumption by comparing them to the case where technological change and trade are treated exogenously. In these cases productivity improvements through technological change and export opportunities are restricted to BAU levels, limiting the systems ability to adjust to increased biofuel demand. Specifically, we evaluate three scenarios - Mix3070, Mix1090, and AllSugarjuice – used as benchmark cases for sensitivity analysis. These scenarios are extended with the assumption of exogenous trade and technological change, denoted as Mix3070Exo Mix1090Exo, and Mix1090Exo respectively, while keeping all other assumptions consistent with their respective endogenous scenarios.

Without technology and trade adjustments, the pressure on domestic resources like land, water, and fertilizer increases significantly. Scenarios with exogenous technological change and trade show substantial increase in cropland, water use, and fertilizer application. In terms of land use, additional cropland requirements increase substantially. Under the Mix3070Exo scenario, 30 million hectares (Mha) of additional land are needed in 2030, reducing slightly to 26 Mha by 2050 compared to the Mix3070 scenario. Similarly, the Mix1090Exo scenario requires 9 Mha in 2030 and 19 Mha in 2050. In contrast, the AllSugarjuiceExo scenario shows only a modest increase of 2 Mha by 2050 compared to the AllSugarjuice scenario, reflecting relatively limited land use pressure in this case.

Water use patterns demonstrate contrasting trends. For the Mix3070Exo scenario, water use decreases by 7 km³ yr ⁻ ¹ in 2030 but rises by 8 km³ yr ⁻ ¹ in 2050 compared to the Mix3070 scenario. The Mix1090Exo scenario shows significant increases in water use, 224 km³ yr ⁻ ¹ in 2030 and 67 km³ yr ⁻ ¹ in 2050, compared to the endogenous scenario. The AllSugarjuiceExo scenario experiences a 5 km³ yr ⁻ ¹ reduction in 2030 but sees a dramatic increase of 249 km³ yr ⁻ ¹ in 2050, indicating potential long-term water stress.

Fertilizer use follows varied trends. The Mix3070Exo scenario sees a slight reduction of 9 Mt yr ⁻ ¹ by 2050 compared to the Mix3070 scenario. In contrast, fertilizer use under the Mix1090Exo scenario rises by 1.8 Mt yr ⁻ ¹ in 2030 and 2.5 Mt yr ⁻ ¹ in 2050, while the AllSugarjuiceExo scenario shows larger increases of 4 Mt yr ⁻ ¹ in 2030 and 4.5 Mt yr ⁻ ¹ in 2050 compared to the AllSugarjuice scenario. This highlights the unsustainable levels of resource intensification required to meet biofuel mandates domestically, which can lead to further environmental degradation. The increased use of cropland and fertilizers under exogenous scenarios results in higher GHG emissions, particularly from land-use changes and fertilizer application. Land use change emissions decrease under the Mix3070Exo scenario, with reductions of 82 Mt CO₂e yr ⁻ ¹ in 2030 and 4 Mt CO₂e yr ⁻ ¹ in 2050. However, land use change emissions rise in the Mix1090Exo and AllSugarjuiceExo scenarios, increasing by 31 Mt CO₂e yr ⁻ ¹ and 2 Mt CO₂e yr ⁻ ¹, respectively, in 2030. Fertilizer-related emissions under the Mix3070Exo scenario decrease by 35 Mt CO₂e yr ⁻ ¹ in 2030 and 132 Mt CO₂e yr ⁻ ¹ in 2050. Conversely, emissions increase in the Mix1090Exo and AllSugarjuiceExo scenarios, rising by 29 Mt CO₂e yr ⁻ ¹ and 26 Mt CO₂e yr ⁻ ¹ in 2030, and further increasing by 9 Mt CO₂e yr ⁻ ¹ and 29 Mt CO₂e yr ⁻ ¹ in 2050, respectively. This indicates that biofuel mandates could exacerbate climate change impacts if not supported by sustainable practices and international collaboration.

## 4 Discussion

This study evaluates biofuel pathways in the context of India’s Biofuel Policy 2018, examining its potential impacts and trade-offs for diverse environmental factors. While biofuels hold promise, their unintended effects on land use, GHG emissions, food security, and water and fertilizer use need thorough long-term evaluation to guide well-informed policy decisions. Our analysis highlights the complexity of achieving the blending mandates, particularly when considering the long-term environmental impacts of the evaluated ethanol production pathways. To meet biofuel blending targets, increased ethanol production necessitates changes in existing cropping patterns or agricultural expansion into other land categories, causing direct and indirect land use change. Our results highlight that ethanol production that is heavily reliant on molasses leads to significant additional land requirements. These demands diminish as the share of molasses decreases and sugarcane juice use increases. Notably, higher molasses use results in cropland expansion into areas with natural vegetation and pasture lands, echoing findings from Schaldach et al. [48] and Lapola et al. [49] about biofuel-driven encroachment on natural areas. However, the extent and intensity of such changes depend on the availability of potential land for expansion [50]. While the exclusive use of sugarcane in the ‘AllSugarjuice’ scenario mitigates some of the environmental impacts compared to exclusively molasses-based production, the increase in fertilizer usage and nitrogen surplus remains concerning. Without effective measures to address these challenges, the long-term sustainability of ethanol production and sugarcane cultivation in India could be jeopardized.

India’s NDCs require additional forest cover by 2030 and restrict deforestation, making agricultural intensification critical to minimize land-use trade-offs. While sugarcane high yield per hectare reduces overall land needs compared to other biofuel crops, indirect land-use changes remain a concern, potentially displacing other crops into forested areas [51]. This highlights the need for policies promoting production intensification, flexible trade regulations, and sustainable agricultural practices.

Water use emerged as a critical challenge in our analysis. Sugarcane, a water-intensive crop, already accounts for a disproportionate share of agricultural water use in India [52]. Our findings indicate that ethanol production relying solely on molasses requires more water compared to mixed scenarios or those dominated by sugarcane juice. Utilizing sugarcane juice directly for ethanol can reduce water consumption, aligning with studies by Lee et al. [53]. However, balancing water-intensive sugarcane cultivation with regional water availability remains imperative. Fertilizer use also presents a significant environmental challenge. High reliance on molasses increases nitrogen fertilizer application, contributing to nutrient runoff and eutrophication [54]. Reducing molasses dependence and incorporating sugarcane juice lowers fertilizer demands and associated emissions. Adoption of sustainable practices, such as precision farming and organic fertilizers, could mitigate these impacts while supporting productivity gains.

The results underscore that achieving the blending mandates will necessitate a 5-to-12-fold increase in sugarcane acreage by 2050 compared to the BAU scenario. Although yield improvements through agricultural intensification could offset some of this requirement, stagnating sugarcane productivity and the environmental costs of increased fertilizer use, particularly nitrogen-based fertilizers, raise critical concerns. High nitrogen application not only elevates greenhouse gas emissions but also risks water quality through nutrient runoff, contributing to eutrophication in nearby water bodies. Moreover, an ethanol policy overly reliant on sugarcane risks converting it into a de facto energy crop, diverting land from food production, and raising concerns over food security. Although the government allowance for direct sugar-to-ethanol conversion has alleviated market surpluses, the pathway’s long-term sustainability remains uncertain without diversified feedstocks.

Economic and market dynamics further complicate biofuel expansion. Scenarios heavily reliant on molasses exacerbate domestic sugar surplus production, destabilizing prices and impacting farmer livelihoods. In contrast, increasing sugarcane juice utilization reduces overproduction, stabilizing sugar markets and enhancing economic outcomes. Our findings suggest that diversification of feedstocks and technological advancements are essential to balance market stability, food security, and environmental sustainability.

The sensitivity analysis underscores the pivotal role of technological progress and international trade in mitigating environmental impacts. Yield improvements must outpace business-as-usual scenarios to meet growing domestic demands while limiting biofuel-induced trade-offs. Sustainable innovation and policy coherence are crucial to achieving biofuel targets without exacerbating environmental degradation or resource competition. Second-generation feedstocks, such as agricultural residues, offer a more sustainable alternative, reducing pressure on land and water resources while mitigating market volatility. However, scaling up second-generation biofuel technologies requires significant policy and infrastructural support.

Future research should broaden the analysis beyond sugarcane-based ethanol to evaluate a more diversified biofuel portfolio, including second-generation feedstocks such as agricultural residues, energy grasses, and municipal organic waste, which can significantly reduce land, water, and fertilizer pressures. Further work is needed to assess regional differences in water availability, groundwater stress, and crop suitability to inform spatially targeted and resource-efficient ethanol production strategies. Examining long-term socio-economic implications such as farm income stability, rural employment, and distributional impacts alongside environmental outcomes will also be essential for designing an equitable and resilient biofuel transition. Additionally, integrating more detailed representations of technological change, irrigation efficiency, and trade policies can help refine pathway assessments and support stronger, evidence-based policy planning for India’s evolving biofuel sector.

## 5 Conclusions

Biofuel production offers a pathway to decarbonisation, but sugarcane-based feedstocks create notable trade-offs. Molasses-based ethanol, while using existing by-products, increases the pressures on land, water, and fertilizer use, raising environmental and economic concerns. Sugarcane juice as a feedstock can ease some of these stresses by addressing sugar surpluses, stabilising prices, and providing farmers with additional income. Meeting India’s New Biofuel Policy targets will require sustainable practices, technological improvement, and adaptive policymaking. Precision agriculture, efficient irrigation, and circular economy approaches such as converting industrial by-products into organic fertilizers can reduce resource burdens. Diversifying feedstocks, including second-generation biomass, will also be essential. Ultimately, success will depend upon policies that balance sustainability with agricultural resilience and long-term economic stability.

## Supporting information

S1 File Additional Information on Method and Results. (DOCX)

S2 File Supplementry Information Data. (XLSX)

S3 File Inclusivity-in-global-research-questionnaire. (DOCX)
