# Supplementary material for: India’s biofuel blending policy presents serious trade-offs with land use, nitrogen emissions and food security
Source: PLoS One. 2026 Jul 8;21(7):e0351419. doi: 10.1371/journal.pone.0351419 (PMC13345272; doi:10.1371/journal.pone.0351419)
Supplement: S1 File — (DOCX) [file pone.0351419.s001.docx]

**Supplementary Information (SI): The Land Use and Environmental Trade-offs of India's Ethanol Blending Target**

1. **Model Description**

The Model of Agricultural Production and its Impact on the Environment (MAgPIE) is a global recursive dynamic partial equilibrium model used to assess land use allocation and competition for resources like water, and the associated consequences for sustainable development under future scenarios of rising food, energy and material demand, climate change impacts, and land-related greenhouse gas mitigation policies (Dietrich et al 2019). The model incorporates geographically explicit information on biophysical conditions (e.g., carbon densities of different vegetation types, agricultural productivity such as crop yields, water availability for irrigation, etc.) into an economic decision-making process (Lotze-Campen et al 2008; Bondeau et al 2007, Müller and Robertson 2014), information of which is obtained from the Lund-Potsdam-Jena managed land model (LPJmL) with a spatial resolution of 0.5°×0.5°. For computational ease, a clustering algorithm is used to aggregate all model inputs in 0.5-degree resolution to simulation units for the optimization process (Dietrich et al 2013).

The model accounts for regional economic conditions such as, elastic demand for agricultural commodities, technological growth, and costs of production. Land types included in MAgPIE are cropland, pasture area, forests, other land (including non-forest natural vegetation, abandoned agricultural land and deserts) and settlements. Cropland (rainfed and irrigated), pasture, forest and other land are endogenously determined, while settlement areas are assumed to be constant over time. Different crop types (e.g., temperate, and tropical cereals, maize, rice, oilseeds, roots), under both rainfed and irrigated systems, and two 2nd generation bioenergy crop types (grassy and woody) are grown on croplands. Cropland can be converted into rangeland, and vice versa. Demand for additional land for meeting increased demand due to policy changes, can be met through a pool of non-agricultural land, but at additional costs. Considering international trade based on historical trade patterns and economic competitiveness, global production must meet demand for food, feed, seed, processing and bioenergy. Population growth and dietary transitions are the main drivers of food demand in the model, which also accounts for changes in intake and food waste, the shift in the share of animal calories, processed products, fruits and vegetables as well as staples.

Flows of greenhouse gases (GHGs) from land use and land-use change are estimated within the MAgPIE model. CO2 emissions are calculated based on changes in carbon stocks of vegetation, which are subject to land-use change dynamics such as conversion of forest into agricultural land (Popp et al 2014). In case of afforestation or when agricultural land is set aside from production, regrowth of natural vegetation absorbs carbon from the atmosphere (negative CO2 emissions). Nitrogen emissions are estimated based on nitrogen budgets for croplands, pastures and the livestock sector (Bodirsky et al 2014). CH4 emissions are based on livestock feed and rice cultivation areas (Popp et al 2010). In the model, pricing of GHG emissions affects the decision-making on land-use and land expansion in the mitigation pathways.

**Fig S1: Model Drivers under the different scenario such as: Population*,* GDP*,* Food Demand, Feed Demand**


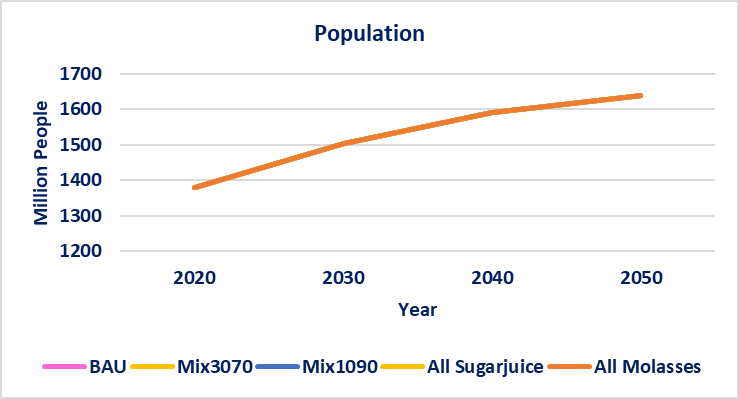

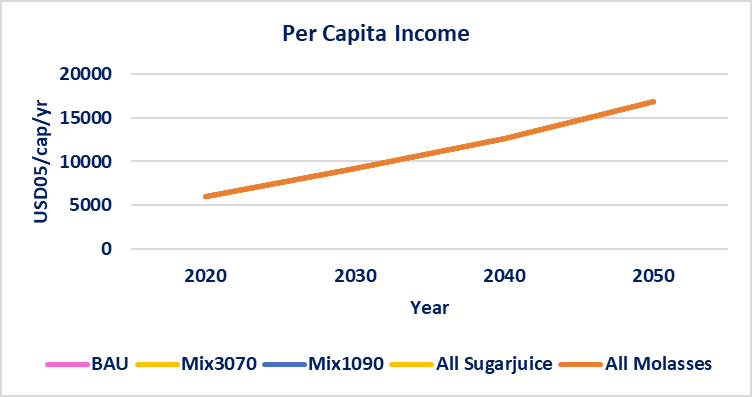


Figure S2. Projected Demand for 1^st^ Generation Bioenergy and Bioethanol

Figure S3. Parameterization of the process to produce ethanol from molasses and sugar juice within the MAgPIE model. The right-hand side of the figure illustrates the process of obtaining molasses from sugarcane and subsequently producing ethanol from molasses. In the MAgPIE model, molasses is treated as a byproduct of sugar production during the refining process, with ethanol being produced from molasses through distillation. The left-hand side of the figure depicts the process of directly producing ethanol from sugarcane juice. All conversion rates are based on the potential yield derived from one tonne of dry matter (tDM) of the primary product when processed into one tDM of the secondary product

**
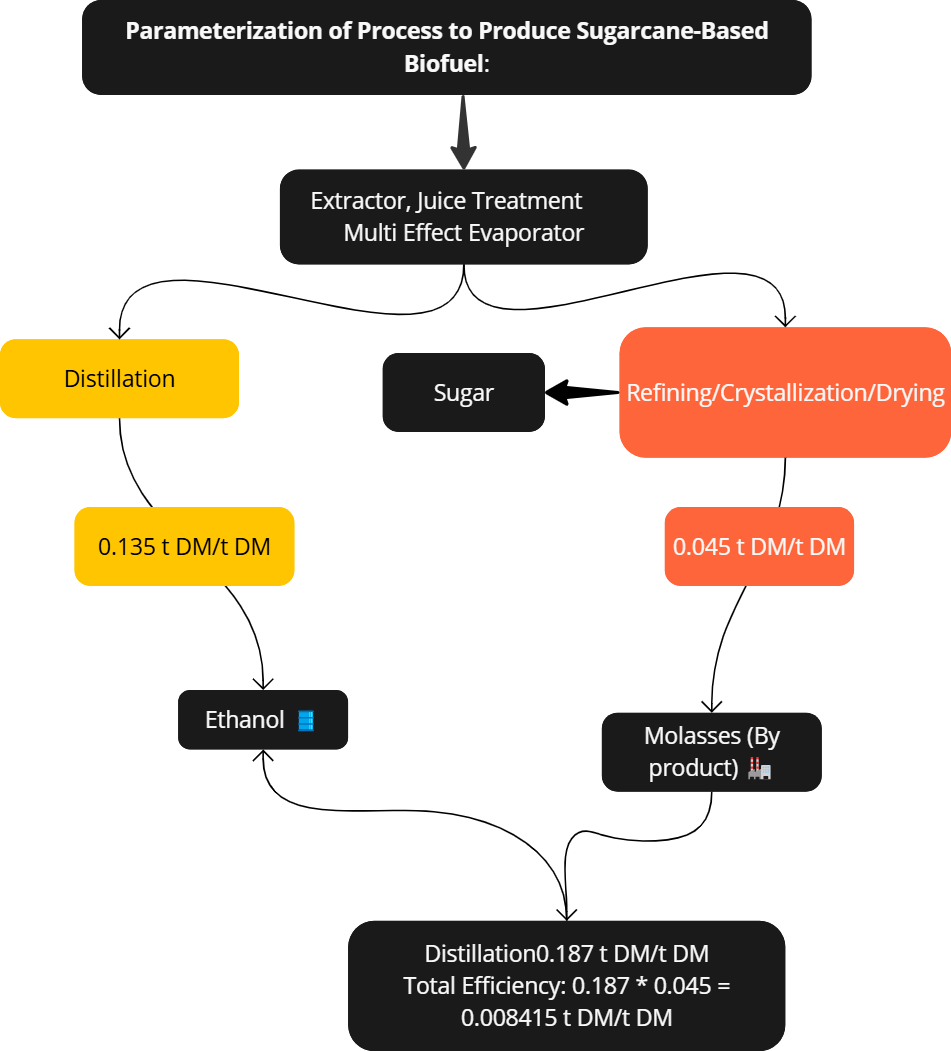
**

Figure S4. the projected increase in technological advancements (Index) and crop productivity (t DM/ha) across scenarios up to 2050, reflecting the intensification of agricultural production systems over time.


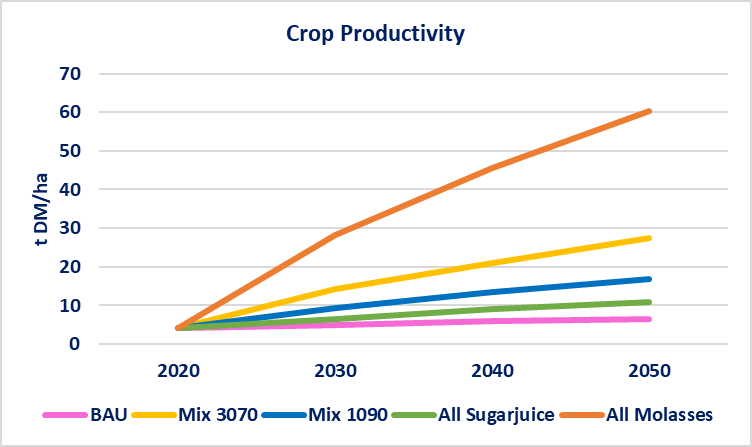

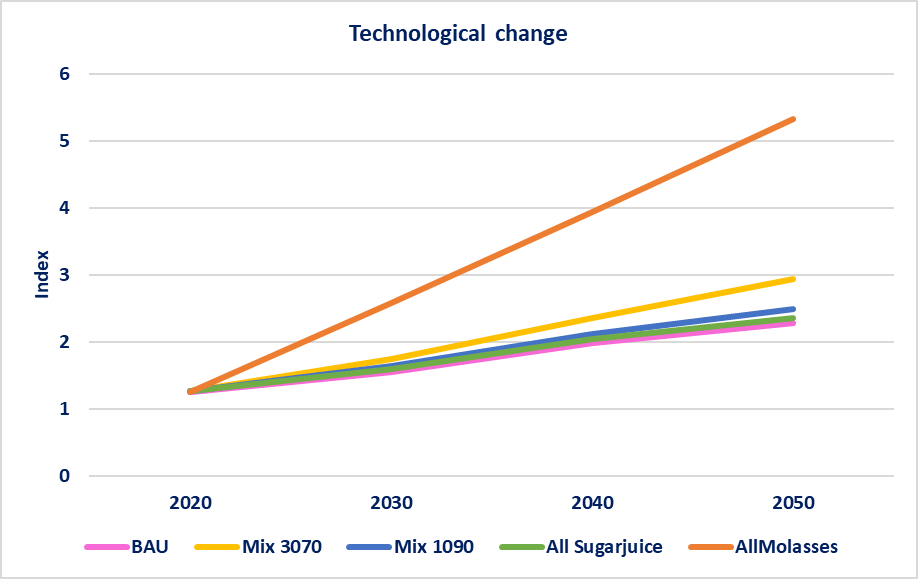


**Figure S5. Projected sugarcane area and production for Sugarcane across scenarios by 2050**

**Figure S6: Crop yield-Irrigated and Rainfed across scenario by 2050**
